# Presence of an *in situ* component is associated with reduced biological aggressiveness of size-matched invasive breast cancer

**DOI:** 10.1038/sj.bjc.6605655

**Published:** 2010-04-27

**Authors:** H Wong, S Lau, T Yau, P Cheung, R J Epstein

**Affiliations:** 1 Division of Haematology and Medical Oncology, Department of Medicine, Queen Mary Hospital, The University of Hong Kong, Hong Kong, China; 2 Oncology Centre and Breast Care Centre, Hong Kong Sanatorium and Hospital, Hong Kong, China

**Keywords:** breast neoplasms, pre-invasive, intraductal, Ki-67, carcinogenesis, tumour progression

## Abstract

**Background:**

The metastatic propensity of invasive ductal carcinoma (IDC) of the breast correlates with axillary node involvement and with expression of the proliferation antigen Ki-67, whereas ductal carcinoma *in situ* (DCIS) is non-metastasising. To clarify whether concomitant DCIS affects IDC prognosis, we compared Ki-67 expression and node status of size-matched IDC subgroups either with DCIS (IDC-DCIS) or without DCIS (pure IDC).

**Methods:**

We analysed data from 1355 breast cancer patients. End points were defined by the association of IDC (with or without DCIS) with grade, nodal status, Ki-67, and ER/HER2.

**Results:**

Size-matched IDC-DCIS was more likely than pure IDC to be screen detected (*P*=0.03), to occur in pre-menopausal women (*P*=0.002), and to be either ER-positive (*P*=0.002) or HER2-positive (*P*<0.0005), but less likely to be treated with breast-conserving surgery (*P*=0.004). Grade and Ki-67 were lower in IDC-DCIS than in pure IDC (*P*=0.02), and declined as the DCIS enlarged (*P*<0.01). Node involvement and lymphovascular invasion in IDC-DCIS increased with the size ratio of IDC to DCIS (*P*<0.01). A 60-month cancer-specific survival favoured IDC-DCIS over size-matched pure IDC (97.4 *vs* 96.0%).

**Conclusion:**

IDC co-existing with DCIS is characterised by lower proliferation and metastatic potential than size-matched pure IDC, especially if the ratio of DCIS to IDC size is high. We submit that IDC-DCIS is biologically distinct from pure IDC, and propose an incremental molecular pathogenesis of IDC-DCIS evolution involving an intermediate DCIS precursor that remains dependent for replication on upstream mitogens.

Widespread use of breast cancer screening with mammography has led to a steep rise in the incidence of ductal carcinoma *in situ* (DCIS; van Steenbergen *et al*, 2009), whereas the overall incidence of invasive ductal carcinoma (IDC) is decreasing (Jemal *et al*, 2009). Although DCIS is pre-malignant (Pinder and Ellis, 2003), the modest improvement in breast cancer mortality attributable to such screening (Mandelblatt *et al*, 2009; Nelson *et al*, 2009) has suggested that many DCIS cases never progress to invasive disease, raising questions about the contribution of DCIS to the natural history of sporadic IDC (Allred *et al*, 2001; Burstein *et al*, 2004). In tumours containing both IDC and DCIS (IDC-DCIS), it is unclear whether the IDC component arises directly from DCIS; in tumours lacking DCIS, however, it is assumed that IDC arises *de novo*. One recent study implicated DCIS as the precursor of IDC-DCIS based on concordant expression of immunohistochemical markers (Steinman *et al*, 2007), a conclusion that has since been supported by genomic data (Aubele *et al*, 2000; Alexe *et al*, 2007; Iakovlev *et al*, 2008), in turn creating a quest for biomarkers that predict invasive transformation of DCIS (Schuetz *et al*, 2006; Castro *et al*, 2008). Other studies have reported differences between DCIS and IDC-DCIS, suggesting that DCIS may not have been a precursor of the invasive component (Farabegoli *et al*, 2002); however, some of these studies have been limited either by failure to use pure IDC as a comparator (Patla *et al*, 2002) or else by small cohort sizes (Mylonas *et al*, 2005). Yet another study concluded that the presence of DCIS was associated with a trend towards superior disease-free survival (DFS) and overall survival, but that it was not an independent predictor of outcome (Chagpar *et al*, 2009).

Proliferation antigen Ki-67 (Gerdes *et al*, 1991) is a biomarker of tumour activity, and an adverse prognostic factor in patients with IDC (Fitzgibbons *et al*, 2000; Jung *et al*, 2009). Elevated Ki-67 expression may predict progression to cancer in some precursor lesions (Shaaban *et al*, 2002); in DCIS, high Ki-67 expression levels may predict *in situ* or invasive recurrence (Simpson *et al*, 2007). Indeed, together with lymph node status, Ki-67 has been implicated as one of only two factors with an independent prognostic value for survival (Jung *et al*, 2009); in tumours not stratified for DCIS status, Ki-67 expression appears prognostically independent of tumour size and nodal status (Barnard *et al*, 1987; Bouzubar *et al*, 1989; Locker *et al*, 1992). Because presence of DCIS may bias the detectability of an associated IDC lesion, we chose in this study to focus on the biological characteristics – Ki-67, nodal status, and receptor expression – of similarly sized IDC with or without DCIS.

## Patients and methods

### Patients

Tumour data obtained from 2271 consecutive patients undergoing surgery for primary invasive breast cancer between October 2000 and September 2008 in a single centre were analysed retrospectively. Male patients (*n*=2), patients with histopathological diagnoses other than ductal carcinoma, such as lobular carcinoma (*n*=75), those who had received neoadjuvant chemotherapy (*n*=56), those with metastatic disease (*n*=71) and samples with incomplete pathological examination or data (*n*=712) were excluded.

### Tumour morphology

The remaining evaluable samples numbered 1355. They were divided into three subgroups based on the association of IDC with DCIS or otherwise, into pure IDC, IDC associated with co-existing DCIS (IDC-DCIS), and pure DCIS. Within the group of IDC-DCIS, the samples were further classified into (1) small IDC–large DCIS, (2) large IDC–small DCIS, and (3) IDC-DCIS with comparable sizes, based on respective median sizes of IDC (1.8 cm) and DCIS (1.5 cm) as the cut-off values. The three subgroups were thus defined to be (1) small IDC (<1.8 cm) large DCIS (⩾1.5 cm), (2) large IDC (⩾1.8 cm) small DCIS (<1.5 cm), and (3) all remaining IDC-DCIS samples. The IDC to DCIS size ratio for each tumour sample was also determined. Sizes of DCIS within IDC-DCIS tumours were initially reported either in terms of largest diameter (cm/mm) or else as a percentage of IDC size; in the latter group, DCIS diameters were calculated from the IDC diameters and rounded to the nearest decimal place. Tumours with DCIS reported to be <1% IDC size were defined for this study as pure IDC.

### Immunohistochemistry

Immunohistochemistry was performed on formalin-fixed paraffin-embedded specimens in a reference laboratory by a dedicated pathologist followed a standard protocol. Immunohistochemistry for ER and PR was performed using 6F11 and 1A6 antibodies respectively, and detected by the polymer EnVision system (Dako, Glostrup, Denmark). ER/PR results were reported with a semi-quantitative *H*-score ranging from 0 to 300; a score above 10 was considered positive. The immunohistochemistry assay used for HER2 was A0485 (polyclonal antibody; Dako). HER2 was considered positive with either 3+ immunoreactivity or amplification by fluorescent *in situ* hybridisation (FISH) with a ratio of HER2 to chromosome 17 centromeric region >2.2, using PathVysion Vysis FISH (Abbott, Chicago, IL, USA). Expression of Ki-67 was assessed by immunostaining with antibody SP6, and was reported as the percentage of positive tumour cells. In cases of IDC-DCIS, Ki-67 values of the invasive cancer component alone were used for comparative analyses.

### Statistical analysis

The median test was used to calculate statistical differences in the Ki-67 index between subgroups; if a difference was found, Spearman's correlation statistics were used to define the relationship between these co-variables. When comparing IDC-DCIS and pure IDC, partial correlations were calculated by controlling for sizes of the invasive cancer component.

### Nodal status, lymphovascular invasion, and outcomes

The number of involved nodes, and the presence or absence of lymphovascular invasion, was determined by routine haematoxylin–eosin staining. Patient DFS were ascertained using Kaplan–Meier methodology.

## Results

### Patients, demographics, histology, and mode of primary tumour detection

The median age of the 1355 subjects was 48 years (range 24–91 years). All were women; most (64.3%) were pre-menopausal, consistent with local epidemiology. Of all tumour samples, 616 (45.5%) were IDC-DCIS; 543 (40.1%) were pure IDC; and the remaining 196 (14.4%) were pure DCIS. Screening mammography detected breast lesions in 169 cases (12.5%), whereas 1036 (76.5%) were self-discovered; 5.7 and 11.5% of patients with IDC and IDC-DCIS, respectively, were diagnosed by screening mammography, whereas 34.2% of patients with pure DCIS were diagnosed by this mode (data not shown). After correcting for IDC size, a partial correlation coefficient of −0.091 (*P*=0.003) favoured pure IDC self-detection and IDC-DCIS screen detection; these data may be pertinent to future cost-efficacy analyses related to screening. Breast-conserving surgery was more common in pure IDC than in IDC-DCIS (47.3 *vs* 39.0%, *P*=0.004), however, implying that more extensive DCIS lesions tended to favour mastectomy despite the higher frequency of screen detection in this group (Table 1).

### Tumour size and proliferation rate

**Figure 1:**
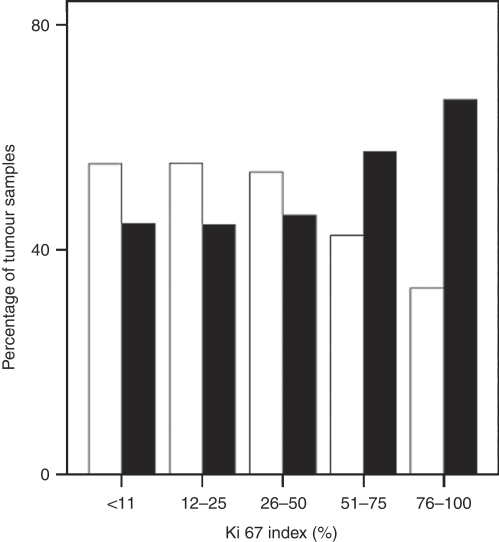
Percentage of invasive ductal carcinoma (IDC) with and without ductal carcinoma *in situ* (DCIS) with different Ki-67 levels. The higher the Ki-67 level, the larger the proportion of pure IDC, and *vice versa* for IDC-DCIS. Black bars, pure IDC; white bars, IDC-DCIS.

As shown in Table 2, median sizes of IDC and DCIS were 1.8 and 1.5 cm (range 0.01–10.0 and 0.1–10.0 cm) respectively. Median Ki-67 levels were higher in pure IDC, compared with IDC-DCIS of comparable invasive size, as determined by the one-tailed median test (*P*=0.03). There was also an overall difference between IDC±DCIS and increasing Ki-67 levels, such that pure IDC was associated with higher Ki-67 than was IDC-DCIS (Spearman's correlation coefficient 0.07, *P*=0.02). After controlling for the sizes of the invasive component, the partial correlation coefficient was still significant at 0.08 (*P*=0.01). In addition, more patients with pure IDC had a higher Ki-67 expression (Figure 1). Analyses on other subgroups were concordant: IDC-DCIS comprised large IDC (⩾1.8 cm) with small DCIS (<1.5 cm) correlated with higher Ki-67 levels than IDC-DCIS with small IDC (<1.8 cm) and large DCIS (⩾1.5 cm), which in turn had higher Ki-67 levels than pure DCIS (correlation coefficient, −0.2; *P*<0.005). Furthermore, higher IDC to DCIS size ratio was associated with higher Ki-67 (Pearson's correlation coefficient, 0.125; *P*=0.002).

### Receptor expression profile

Compared with pure IDC, IDC-DCIS tumours were more often ER positive (81.5 *vs* 74.0%, *P*=0.002), PR positive (74.7 *vs* 70.5%, *P*=0.114), and/or HER2 positive (25.5 *vs* 16.2%, *P*<0.0005). These IDC-DCIS trends were similar in pure DCIS (Table 3), supporting the view that the IDC-DCIS phenotype reflects that of the associated (presumably precursor) DCIS. Moreover, as HER2 expression and ER expression tend to be inversely related, this dual finding suggests a broader dependence of IDC-DCIS on mitogenic signalling.

### Metastasis and survival

Table 4 shows that most patients (66.4%) were free of nodal involvement; 301 (22.2%) had 1–3 involved lymph nodes, whereas 137 (10.1%) had 4 or more involved nodes. Within the 616 IDC-DCIS samples, the mean value of IDC to DCIS size ratio was 5 (range 0.025–50). There was a trend towards higher number of involved lymph nodes in pure IDC compared with IDC-DCIS (Pearson's correlation coefficient, 0.052; *P*=0.078). Within the IDC-DCIS group, higher IDC to DCIS size ratio correlated with more involved lymph nodes (Pearson's correlation coefficient, 0.112; *P*=0.006). Similarly, IDC-DCIS cases with large IDC–small DCIS were more often associated with lymphovascular invasion (57.7%) than were small IDC–large DCIS (27.0% *P*<0.0005); this finding was confirmed by the correlation between higher IDC-DCIS size ratio with lymphovascular invasion (Spearman's correlation coefficient, 0.163, *P*<0.0005). With a median follow-up of 29.3 months, the 5-year DFS rates of the IDC-DCIS and pure IDC subgroups were 97.40 and 96.00% respectively (not significant; *P*=0.38).

## Discussion

Breast cancer is a heterogenous disease, and much research has been directed towards identifying subtypes to aid risk stratification. The paradigm of early breast cancer management is thus shifting towards ‘personalising’ therapy as a function of morphological, biological, and molecular disease variables (Epstein, 2009). *In situ* cancer – especially DCIS – has attracted much interest because of its rising incidence in the mammography era, but there are few studies scrutinising the significance of IDC co-existing with DCIS (Chagpar *et al*, 2009). Currently, the association of DCIS in IDC has no bearing on systemic treatment, which depends entirely on the pathological and molecular characteristics of IDC. It therefore remains unclear whether IDC-DCIS behaves identically to pure IDC or otherwise, notwithstanding that this distinction could have significant implications for adjuvant treatment in early breast cancer.

This study reports the largest cohort yet published relating to this issue, showing that the extent of DCIS with IDC correlates with lower Ki-67 index and fewer involved lymph nodes. Although the pre-malignant DCIS may well have permitted earlier detection of the IDC component than would otherwise occur for a pure IDC of similar size, the fact that the proliferative and metastatic differences persist after controlling for IDC size supports the impression of a biological difference between IDC-DCIS and pure IDC. This conclusion is further supported by the finding of increased ER/PR and/or HER2 expression in IDC-DCIS, suggesting in turn that the pure IDC carcinogenesis pathway could favour the therapeutically challenging basaloid phenotype, which is already known to be associated with multiple defects in tumour suppressor genes such as *TP53* and the *BRCA* DNA repair genes.

**Figure 2:**
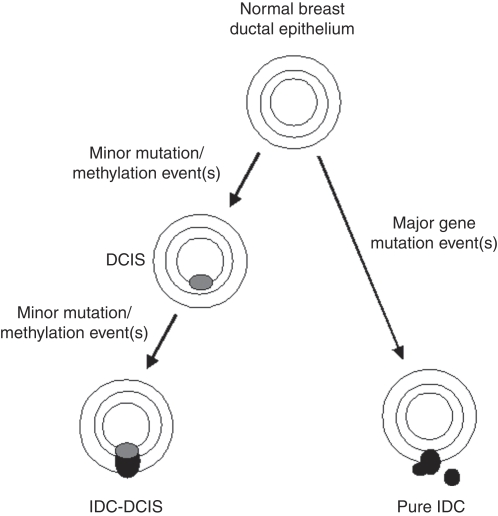
Hypothetical carcinogenesis model for invasive ductal carcinoma–ductal carcinoma *in situ* (IDC-DCIS) *vs* pure IDC. In this model, IDC-DCIS – intrinsically less aggressive as reflected by lower Ki-67 expression and less nodal involvement – develops stepwise from normal breast epithelium via DCIS, through acquiring minor sequential genetic dysfunctions each time, whereas pure IDC develops *de novo* due to major genetic event(s).

These results imply that IDC-DCIS tends to have lower disease aggressiveness than pure IDC, particularly as DCIS-IDC size ratios increase. Such a model is reminiscent of colorectal cancer that may arise either through a polyp-dependent (adenoma carcinoma) process, or through a non-polyp-dependent (*de novo*) pathway associated with greater invasive and metastatic potential (Tanaka, 2009). In preclinical models of breast cancer, disruption of the TP53 pathway and/or constitutive activation of HER2, FAK or KRAS produce DCIS, but further inhibition of pRb signalling and/or activation of the phosphatidylinositol 3′-kinase pathway appear necessary to promote malignant progression (Lightfoot *et al*, 2004; Golubovskaya *et al*, 2009; Wu *et al*, 2009). Comparative genomic hybridisation studies have also identified genetic pathways – for example through loss of genetic material of 16q associated with well-differentiated to intermediately differentiated DCIS and grade 1 IDC, or gains of 8q, 17q, and 20q associated with poorly differentiated DCIS and grade 3 IDC (Buerger *et al*, 2001) – that support a linear progression model for the transition from normal to DCIS to IDC. However, the heterogeneity of genetic changes in DCIS compared to IDC suggests other breast cancer progression pathways (Gao *et al*, 2009). Hence, given that fast-replicating (high Ki-67) tumours might be expected to contain a higher burden of dysfunctional tumour suppressor genes, our study results raise the possibility that pure IDC tumours arise as a result of more drastic tumour suppressor gene defects, whereas IDC-DCIS tumours tend to evolve as a result of a more incremental accumulation of milder suppressor gene defects (Figure 2).

We acknowledge that this study has several important limitations. First, it is a retrospective analysis, from which almost one-third of case records originally examined were excluded due to incomplete data. Second, there were variations in pathology reporting styles, as the tumour samples were not centrally reviewed. Third, the median follow-up time of 30 months is too short to expect major differences in disease-free survival to have emerged. Fourth, it must remain speculative for now whether concomitant DCIS will emerge as a useful independent prognostic variable, as the degree of biological overlap between size-matched IDC-DCIS and pure IDC may preclude this. Finally, we acknowledge that the postulation of molecular differences between IDC-DCIS and pure IDC, as posited in Figure 2, remains entirely speculative at this point, and that any such assumption is premature. We hope, however, that the indirect clinicopathological observations provided here might provoke more specific genetic and scientific hypothesis testing in this clinical context.

In conclusion, this study raises the possibility that the association or otherwise of IDC with a DCIS precursor, and the relative sizes of these two lesions, could be factored into future adjuvant therapeutic decision-making. If verified, this distinction would in turn suggest an as-yet-undetermined molecular basis for the divergent clinical behaviour of these breast lesion subtypes. Future work is needed to test the observations noted here, and to explore molecular explanations for the putative differences in natural history.

## Figures and Tables

**Table 1:** Demographic, detection, and surgical data of patients with pure IDC, IDC-DCIS, and pure DCIS

			**IDC-DCIS**		
**Characteristics**	**All cases**	**Pure IDC**	**All IDC-DCIS**	**Small IDC–large DCIS**	**Large IDC–small DCIS**
*Age at diagnosis*					
Median (range)	48 (24–91)	49 (24–91)	47 (26–86)	47 (26–83)	47 (29–86)
⩽35	7.5 (101)	8.1 (44)	6.7 (41)	7.4 (14)	6.8 (15)
36–50	53.5 (726)	47.5 (258)	58.1 (358)	60.3 (114)	54.5 (121)
⩾51	38.5 (521)	43.3 (235)	35.2 (217)	32.3 (61)	38.7 (86)
Unknown	0.5 (7)	1.1 (6)	0.0 (0)	0.0 (0)	0.0 (0)
					
*Menopausal status*					
Pre-menopausal	64.3 (871)	58.6 (318)	67.2 (414)	70.4 (133)	62.6 (139)
Post-menopausal	35.7 (484)	41.4 (225)	32.8 (202)	29.6 (56)	37.4 (83)
					
*Mode of discovery*					
Mammographic screening	12.5 (169)	5.7 (31)	11.5 (71)	18.5 (35)	4.5 (10)
Self-detected	76.5 (1036)	82.7 (449)	77.4 (477)	68.8 (130)	86.9 (193)
Others	11.1 (150)	11.6 (63)	11.0 (68)	12.7 (24)	8.6 (19)
					
*Primary surgery*					
Mastectomy and axillary dissection	15.1 (205)	16.0 (87)	18.8 (116)	16.9 (32)	19.4 (43)
Lumpectomy and axillary surgery (including sentinel lymph node biopsy)	40.3 (546)	47.3 (257)	39.0 (240)	22.8 (43)	47.7 (106)
Lumpectomy alone	1.8 (24)	1.5 (8)	0.5 (3)	0.5 (1)	0.0 (0)
Others	42.8 (580)	35.2 (191)	41.7 (257)	59.8 (113)	32.9 (73)
					
Total number	1355	543	616	189	222

Abbreviations: IDC=invasive ductal carcinoma; DCIS=ductal carcinoma *in situ*. Data are presented as percentages, with numerical results following in parentheses.

**Table 2:** Tumour size, grade, and Ki-67 expression in patients with pure IDC *vs* IDC-DCIS

			**IDC-DCIS**		
**Characteristics**	**All cases**	**Pure IDC**	**All IDC–DCIS**	**Small IDC–large DCIS**	**Large IDC–small DCIS**
*IDC size (cm)*					
Median (range)	1.8 (0.01–10.0)	1.8 (0.12–10.0)	1.8 (0.01–8.00)	0.7 (0.01–1.7)	2.5 (1.8–8.0)
⩽2	50.0 (678)	58.0 ((315)	58.9 (363)	100 (189)	15.8 (35)
>2–5	32.6 (442)	37.9 (206)	38.3 (236)	0.0 (0)	79.3 (176)
>5	2.9 (39)	4.1 (22)	2.8 (17)	0.0 (0)	5.0 (11)
					
*DCIS size (cm)*					
Median (range)	1.5 (0.1–10.0)	NA	1.0 (0.1–10.0)	3.4 (1.5–10.0)	0.3 (0.1–1.4)
					
*Grade*					
1	9.4 (128)	8.3 (45)	13.3 (82)	15.3 (29)	12.2 (27)
2	30.9 (419)	35.5 (193)	36.7 (226)	41.6 (77)	32.0 (71)
3	44.1 (598)	54.7 (297)	48.9 (301)	41.8 (79)	55.9 (124)
Unknown	15.5 (210)	1.5 (8)	1.1 (7)	2.1 (4)	0.0 (0)
					
*Ki-67 index* (%)					
Mean, median	21.3, 13.0	25.3, 15.0	21.0, 14.0	19.1, 12.0	23.5, 15.5
⩽10	45.7 (619)	40.1 (218)	44.0 (271)	47.6 (90)	36.0 (80)
>10–20	21.2 (287)	19.7 (107)	22.7 (140)	22.8 (43)	23.9 (53)
>20	33.1 (449)	40.1 (218)	33.3 (205)	29.6 (56)	40.1 (89)
					
Total number	1355	543	616	189	222

Abbreviations: IDC=invasive ductal carcinoma; DCIS=ductal carcinoma *in situ.* Results are presented as percentages, with raw data following in parentheses.

**Table 3:** Comparative receptor expression profile – ER/PR and HER2 protein immunohistochemical status – of pure IDC, IDC-DCIS, and pure DCIS

**Receptor phenotype**	**Pure IDC**	**IDC-DCIS**	**Pure DCIS**
ER-negative	26.0 (141)	18.5 (114)	22.4 (44)
ER-positive	74.0 (402)	81.5 (502)	77.6 (152)
PR-negative	29.5 (160)	25.3 (156)	25.0 (49)
PR-positive	70.5 (383)	74.7 (460)	75.0 (147)
HER2-negative	82.9 (450)	73.2 (451)	65.3 (128)
HER2-positive	16.2 (88)	25.5 (157)	34.7 (68)

Abbreviations: ER=oestrogen receptor; PR=progesterone receptor; IDC=invasive ductal carcinoma; DCIS=ductal carcinoma *in situ.*

Results are presented as percentages, with numerical case data following in parentheses.

**Table 4:** Nodal status and lymphovascular invasion in pure IDC *vs* IDC-DCIS

			**IDC-DCIS**		
**Characteristics**	**All cases**	**Pure IDC**	**All IDC–DCIS**	**Small IDC–large DCIS**	**Large IDC–small DCIS**
*Lymph node status*					
Negative	66.4 (900)	58.9 (320)	62.3 (384)	72.0 (136)	55.4 (123)
1–3 lymph nodes	22.2 (301)	26.7 (145)	25.3 (156)	17.5 (33)	29.7 (66)
4–9 lymph nodes	6.9 (94)	8.7 (47)	7.6 (47)	6.3 (12)	10.8 (24)
>9 lymph nodes	3.2 (43)	5.0 (27)	2.6 (16)	1.1 (2)	3.6 (8)
Unknown	1.3 (17)	0.7 (4)	2.1 (13)	3.2 (6)	0.5 (1)
					
*Lymphovascular invasion*					
Negative	65.2 (884)	60.8 (330)	58.3 (359)	73.0 (138)	42.3 (94)
Positive	34.8 (471)	39.2 (213)	41.7 (257)	27.0 (51)	57.7 (128)
					
Total number	1355	543	616	189	222

Abbreviations: IDC=invasive ductal carcinoma; DCIS=ductal carcinoma *in situ.* Data are presented as percentages, with total case numbers following in parentheses.

## References

[bib1] Alexe G, Dalgin GS, Ganesan S, Delisi C, Bhanot G (2007) Analysis of breast cancer progression using principal component analysis and clustering. J Biosci 32: 1027–1039 17914245 10.1007/s12038-007-0102-4

[bib2] Allred DC, Mohsin SK, Fuqua SA (2001) Histological and biological evolution of human premalignant breast disease. Endocr Relat Cancer 8: 47–61 11350726 10.1677/erc.0.0080047

[bib3] Aubele M, Mattis A, Zitzelsberger H, Walch A, Kremer M, Welzl G, Hofler H, Werner M (2000) Extensive ductal carcinoma *in situ* with small foci of invasive ductal carcinoma: evidence of genetic resemblance by CGH. Int J Cancer 85: 82–86 10585588 10.1002/(sici)1097-0215(20000101)85:1<82::aid-ijc15>3.0.co;2-s

[bib4] Barnard NJ, Hall PA, Lemoine NR, Kadar N (1987) Proliferative index in breast carcinoma determined *in situ* by Ki67 immunostaining and its relationship to clinical and pathological variables. J Pathol 152: 287–295 3668731 10.1002/path.1711520407

[bib5] Bouzubar N, Walker KJ, Griffiths K, Ellis IO, Elston CW, Robertson JF, Blamey RW, Nicholson RI (1989) Ki67 immunostaining in primary breast cancer: pathological and clinical associations. Br J Cancer 59: 943–947 2472168 10.1038/bjc.1989.200 PMC2246720

[bib6] Buerger H, Mommers EC, Littmann R, Simon R, Diallo R, Poremba C, Dockhom-Dwomiczak B, van Diest PJ, Boecker W (2001) Ductal invasive G2 and G3 carcinomas of the breast are the end stages of at least two different lines of genetic evolution. J Pathol 194: 165–170 11400144 10.1002/path.875

[bib7] Burstein HJ, Polyak K, Wong JS, Lester SC, Kaelin CM (2004) Ductal carcinoma *in situ* of the breast. N Engl J Med 350: 1430–1441 15070793 10.1056/NEJMra031301

[bib8] Castro NP, Osorio C, Torres C, Bastos EP, Mourão-Neto M, Soares FA, Brentani HP, Carraro DM (2008) Evidence that molecular changes in cells occur before morphological alterations during the progression of breast ductal carcinoma. Breast Cancer Res 10: R87 18928525 10.1186/bcr2157 PMC2614523

[bib9] Chagpar AB, McMasters KM, Sahoo S, Edwards MJ (2009) Does ductal carcinoma *in situ* accompanying invasive carcinoma affect prognosis? Surgery 146: 561–567 19789013 10.1016/j.surg.2009.06.039

[bib10] Epstein RJ (2009) TNM: therapeutically not mandatory. Eur J Cancer 45: 1111–1116 19328677 10.1016/j.ejca.2009.02.020

[bib11] Farabegoli F, Champeme M, Bieche I, Santini D, Ceccarelli C, Derenzini M, Lidereau R (2002) Genetic pathways in the evolution of breast ductal carcinoma *in situ*. J Pathol 196: 280–286 11857490 10.1002/path.1048

[bib12] Fitzgibbons PL, Page DL, Weaver D, Thor AD, Allred DC, Clark GM, Ruby SG, O'Malley F, Simpson JF, Connolly JL, Hayes DF, Edge SB, Lichter A, Schnitt SJ (2000) Prognostic factors in breast cancer. College of American Pathologists Consensus Statement 1999. Arch Pathol Lab Med 124: 966–978 10888772 10.5858/2000-124-0966-PFIBC

[bib13] Gao Y, Niu Y, Wang X, Wei L, Lu S (2009) Genetic changes at specific stages of breast cancer progression detected by comparative genomic hybridization. J Mol Med 87: 145–152 18936904 10.1007/s00109-008-0408-1

[bib14] Gerdes J, Li L, Schlueter C, Duchrow M, Wohlenberg C, Gerlach C, Stahmer I, Kloth S, Brandt E, Flad HD (1991) Immunobiochemical and molecular biologic characterization of the cell proliferation-associated nuclear antigen that is defined by monoclonal antibody Ki-67. Am J Pathol 138: 867–873 2012175 PMC1886092

[bib15] Golubovskaya VM, Conway-Dorsey K, Edmiston SN, Tse C, Lark AA, Livasy CA, Moore D, Millikan RC, Cance WG (2009) FAK overexpression and p53 mutations are highly correlated in human breast cancer. Int J Cancer 125: 1735–1738 19521985 10.1002/ijc.24486 PMC2773794

[bib16] Iakovlev V, Arneson N, Wong V, Wang C, Leung S, Iokovleva G, Warren K, Pintilie M, Done S (2008) Genomic differences between pure ductal carcinoma *in situ* of the breast and that associated with invasive disease: a calibrated aCGH study. Clin Cancer Res 14: 4446–4454 18628458 10.1158/1078-0432.CCR-07-4960

[bib17] Jemal A, Siegel R, Ward E, Hao Y, Xu J, Thun MJ (2009) Cancer statistics, 2009. CA Cancer J Clin 59: 225–249 19474385 10.3322/caac.20006

[bib18] Jung SY, Han W, Lee JW, Ko E, Kim E, Yu JH, Moon HG, Park IA, Oh DY, Im SA, Kin TY, Hwang KT, Kin SW, Noh DY (2009) Ki-67 expression gives additional prognostic information on St. Gallen 2007 and Adjuvant! Onlin risk categories in early breast cancer. Ann Surg Oncol 16: 1112–1121 19219507 10.1245/s10434-009-0334-7

[bib19] Lightfoot HMJ, Lark A, Livasy CA, Moore DT, Cowan D, Dressler L, Craven RJ, Cance WG (2004) Upregulation of focal adhesion kinase (FAK) expression in ductal carcinoma *in situ* (DCIS) is an early event in breast tumorigenesis. Breast Cancer Res Treat 88: 109–116 15564794 10.1007/s10549-004-1022-8

[bib20] Locker AP, Birrell K, Bell JA, Nicholson RI, Elston CW, Blamey RW, Ellis IO (1992) Ki67 immunoreactivity in breast carcinoma: relationships to prognostic variables and short term survival. Eur J Surg Oncol 18: 224–229 1607032

[bib21] Mandelblatt JS, Cronin KA, Bailey S, Berry DA, de Koning HJ, Draisma G, Huang H, Lee SJ, Munsell M, Plevritis SK, Ravdin P, Schechter CB, Sigal B, Stoto MA, Stout NK, van Ravesteyn NT, Venier J, Zelen M, Feuer EJ, for the Breast Cancer Working Group of the Cancer Intervention and Surveillance Modeling Network (CISNET) (2009) Effects of mammography screening under different screening schedules: model estimates of potential benefits and harms. Ann Intern Med 151: 738–747 19920274 10.1059/0003-4819-151-10-200911170-00010 PMC3515682

[bib22] Mylonas I, Makovitzky J, Jeschke U, Briese V, Friese K, Gerber B (2005) Expression of Her2/neu, steroid receptors (ER and PR), Ki67 and p53 in invasive mammary ductal carcinoma associated with ductal carcinoma *in situ* (DCIS) versus invasive breast cancer alone. Anticancer Res 25: 1719–1723 16033090

[bib23] Nelson HD, Tyne K, Naik A, Bougatsos C, Chan BK, Humphrey L (2009) Screening for breast cancer: an update for the U.S. Preventive Services Task Force. Ann Intern Med 151: 727–737 19920273 10.1059/0003-4819-151-10-200911170-00009 PMC2972726

[bib24] Patla A, Rudnicka-Sosin L, Pawlega J, Stachura J (2002) Prognostic significance of selected immunohistochemical parameters in patients with invasive breast carcinoma concomitant with ductal carcinoma *in situ*. Pol J Pathol 53: 25–27 12014222

[bib25] Pinder SE, Ellis IO (2003) The diagnosis and management of pre-invasive breast disease: ductal carcinoma *in situ* (DCIS) and atypical ductal hyperplasia (ADH) – definitions and classification. Breast Cancer Res 5: 254–257 12927035 10.1186/bcr623 PMC314427

[bib26] Schuetz CS, Bonin M, Clare SE, Nieselt K, Sotlar K, Walter M, Fehm T, Solomayer E, Riess O, Wallwiener D, Kurek R, Neubauer HJ (2006) Progression-specific genes identified by expression profiling of matched ductal carcinomas *in situ* and invasive breast tumors, combining laser capture microdissection and oligonucleotide microarray analysis. Cancer Res 66: 5278–5286 16707453 10.1158/0008-5472.CAN-05-4610

[bib27] Shaaban AM, Sloanne JP, West CR, Foster CS (2002) Breast cancer risk in usual ductal hyperplasia is defined by estrogen receptor-alpha and Ki-67 expression. Am J Pathol 160: 597–604 11839580 10.1016/s0002-9440(10)64879-1 PMC1850641

[bib28] Simpson PT, Da Silva LM, Lakhani SR (2007) *In situ* carcinoma – can we predict which patient will come back with a recurrence? Cancer Cell 12: 409–411 17996643 10.1016/j.ccr.2007.10.026

[bib29] Steinman S, Wang J, Bourne P, Yang Q, Tang P (2007) Expression of cytokeratin markers, ER-alpha, PR, HER-2/neu, and EGFR in pure ductal carcinoma *in situ* (DCIS) and DCIS with co-existing invasive ductal carcinoma (IDC) of the breast. Ann Clin Lab Sci 37: 127–134 17522367

[bib30] Tanaka T (2009) Colorectal carcinogenesis: review of human and experimental animal studies. J Carcinog 8: 5 19332896 10.4103/1477-3163.49014 PMC2678864

[bib31] van Steenbergen LN, Voogd AC, Roukema JA, Louwman WJ, Duijm LE, Coebergh JW, van de Poll-Franse LV (2009) Screening caused rising incidence rates of ductal carcinoma *in situ* of the breast. Breast Cancer Res Treat 115: 181–183 18516674 10.1007/s10549-008-0067-5

[bib32] Wu M, Jung L, Cooper AB, Fleet C, Chen L, Breault L, Clark K, Cai Z, Vincent S, Bottega S, Shen Q, Richardson A, Bosenburg M, Naber SP, DePinho RA, Kuperwasser C, Robinson MO (2009) Dissecting genetic requirements of human breast tumorigenesis in a tissue transgenic model of human breast cancer in mice. Proc Natl Acad Sci USA 106: 7022–7027 19369208 10.1073/pnas.0811785106 PMC2669443

